# Simulation/Experiment Confrontation, an Efficient Approach for Sensitive SAW Sensors Design

**DOI:** 10.3390/s20174994

**Published:** 2020-09-03

**Authors:** Bilel Achour, Ghada Attia, Chouki Zerrouki, Najla Fourati, Kosai Raoof, Nourdin Yaakoubi

**Affiliations:** 1 LAUM, UMR CNRS 6613, Le Mans Université, Avenue Olivier Messiaen, CEDEX 9, 72085 Le Mans, France; bilel.achour@univ-lemans.fr (B.A.); kosai.raoof@univ-lemans.fr (K.R.); 2 Cnam, SATIE, UMR CNRS 8029, 292 Rue Saint Martin, 75003 Paris, France; ghada.attia@lecnam.net (G.A.); fourati@cnam.fr (N.F.)

**Keywords:** finite element method (FEM) simulation, polyisobutylene (PIB), dichloromethane (DCM), anthracene, zinc ions, surface acoustic wave (SAW) sensors, dissociation constants

## Abstract

Sensitivity is one of the most important parameters to put in the foreground in all sensing applications. Its increase is therefore an ongoing challenge, particularly for surface acoustic wave (SAW) sensors. Herein, finite element method (FEM) simulation using COMSOL Multiphysics software is first used to simulate the physical and electrical properties of SAW delay line. Results indicate that 2D configuration permits to accurately obtain all pertinent parameters, as in 3D simulation, with very substantial time saving. A good agreement between calculation and experiment, in terms of transfer functions (S21 spectra), was also shown to evaluate the dependence of the SAW sensors sensitivity on the operating frequency; 2D simulations have been conducted on 104 MHz and 208 MHz delay lines, coated with a polyisobutylene (PIB) as sensitive layer to dichloromethane (DCM). A fourfold increase in sensitivity was obtained by doubling frequency. Both sensors were then realized and tested as chem-sensors to detect zinc ions in liquid media. 9-{[4-({[4-(9anthrylmethoxy)phenyl]sulfanyl} methyl)]methyl] anthracene (TDP-AN) was selected as the sensing layer. Results show a comparable response curves for both designed sensors, in terms of limit of detection and dissociation constants K_d_ values. On the other hand, experimental sensitivity values were of the order of [7.0 ± 2.8] × 10^8^ [°/M] and [16.0 ± 7.6] × 10^8^ [°/M] for 104 MHz and 208 MHz sensors, respectively, confirming that the sensitivity increases with frequency.

## 1. Introduction

Surface acoustic wave (SAW) devices are investigated in a large variety of applications including signal processing [1], mobile and wireless communication [2,3], modulators [4] and RF filters [5]. In the last decades, they have been more and more used as chemical and biological sensors due to their fast response [6], low cost [7], high sensitivity [8,9,10,11,12,13], low limit of detection [14,15] and real-time monitoring [16]. To design and optimize SAW sensors with time and money saving, several simulation methods were developed: delta function model [17], equivalent circuit model [18], P-matrix model [19], coupled mode theory [20], and CST (Computer Simulation Technology) software [21]. However, these methods are very complex and are mainly used in telecommunication applications such as filters and resonators. To overcome these limitations, Finite Element Method (FEM) simulation was investigated and has proven to be the most suitable method for metrological characterization of SAW devices prior to their fabrication in a clean room [22,23,24,25,26].

Sensitivity is one of the most important characteristic parameters of SAW chemical and biological sensors. Several theoretical research and simulations have therefore been conducted to improve sensors sensitivity. Abdoallahi et al. have carried out a 3D-FEM modelling to evaluate SAW devices mass sensitivity by calculating wave propagation speeds and the further energy distributions [27]. Zhao et al. investigated FEM modeling to analyze the polymer coating effects on the surface acoustic waves propagation in the absence and presence of vapor [28]. Richardson et al. have used 3D-FEM simulation to compare insertion loss and mass sensitivity of SAW sensors having microcavities filled with ZnO and nanocrystalline diamond (NCD) [29]. In a recent study, Abraham et al., achieved a significative improvement in sensitivity by functionalizing the SAW’s sensing layer with ZnO-CuO nanocomposites in MOX-CNT nanocomposite [30]. In the present work, we first used MATLAB to rapidly simulate the behavior of a delay line. Among the different models that can be implemented for this kind of system, crossed-field equivalent circuit model, coupling of modes model, impulse response model, we opted for the impulse response one [31]. FEM simulation, using COMSOL Multiphysics software, was then used as it allows to consider material density, particle displacement, and electric potential during the propagation of surface acoustic waves on the surface of a given piezoelectric material. Simulations started first on an existing SAW sensor operating at 104 MHz, by investigating different configurations. Only the one which was in perfect agreement with the experimental results was considered. This optimization phase has permitted to validate the accuracy of the simulations before their subsequent use to estimate the frequency responses of newly designed structures. To simulate the increase in sensitivity with the operating frequency, a polyisobutylene (PIB) layer, sensitive to dichloromethane (DCM) gas, was deposited on the SAWs sensing area. Based on the computational results, a new optimized structure was designed and tested for zinc ions detection. The choice of these ions is related to their presence in Sarthe River Basin of “Le Mans” region [32]. Several receptors have been investigated for zinc ions detection, mainly ion imprinted polymers [33,34,35], DNA [36], aptamers [37], self-assembled monolayers (SAM) [38,39], enzymes [40,41], and molecular cages [42,43,44,45]. Herein, we have chosen to functionalize the sensitive zone of the 104 MHz and 208 MHz SAW sensor with 9-{[4-({[4-(9 anthrylmethoxy) phenyl]sulfanyl} methyl)]methyl] anthracene (TDP-AN). This molecule was chosen among other ones due to its easiness to be synthetized and its capacity to bind to gold surfaces through the S–Au bond. Results were compared in terms of limit of detection (L.O.D), sensitivity, and dissociation constant (K_d_), which is related to the degree of the affinity between the analyte and the investigated sensing layer.

## 2. Materials and Methods

### 2.1. Chemicals

Sulfuric acid (H_2_SO_4_, 98%), hydrogen peroxide (H_2_O_2_, 30%), chloroform (CHCl_3_), and zinc (II) chloride (ZnCl_2_) were purchased from Sigma-Aldrich (St. Louis, MO, USA).

**Scheme 1:**
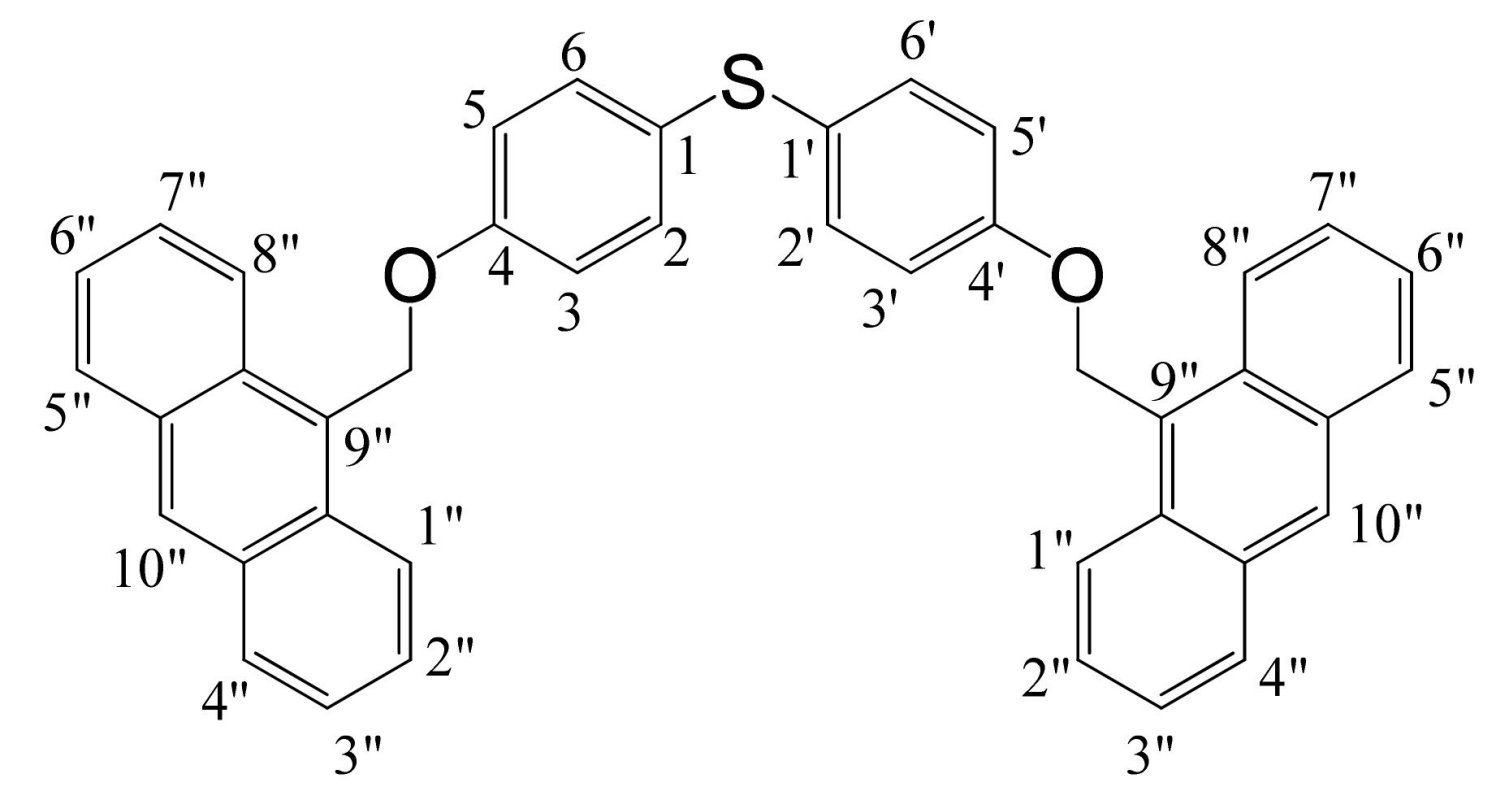
Chemical structure of TDP-AN.

9-{[4-({[4-(9 anthrylmethoxy) phenyl]sulfanyl} methyl)]methyl] anthracene (TDP-AN), whose chemical structure is presented in Scheme 1, was synthetized via the Williamson reaction between 9-chloromethyl anthracene (AnCl) and 4,4′-thiodiphenol.

### 2.2. Gravimetric Measurements

The designed 104 and 208 MHz SAW sensors were fabricated on 36 YX-LiTaO_3_ piezoelectric substrate. Their sensitive area and interdigital transducer electrodes (IDTs) were metallized with 20 nm/80 nm Cr/Au layers. The 36 YX-cut allows mainly to generate two horizontal transverse waves on the surface of the LiTaO3 substrate: surface skimming bulk waves (SSBW) and leaky-SAW. These waves can however coexist with fast shear bulk acoustic waves and quasi-longitudinal bulk acoustic waves. However, and as the SAW sensing area was metallized with 20/80 nm Cr/Au thin layers, LSAW propagation was favored compared to SSBW. Besides, bulk acoustic waves (BAWs) are equally generated with a lesser magnitude and propagate in the material under certain inclination. Their detection is thus possible after backscattering.

To obtain an operating frequency of about 104 and 208 MHz sensors, the IDTs were photolithographically patterned with a periodicity of 40 and 20 µm respectively (See Supplementary Materials Figure S1). The measurement setup consists of (i) a SAW sensor (See Supplementary Materials Figure S2); (ii) a printed circuit board to ensure electrical contacts (See Supplementary Materials Figure S3); (iii) a Kalrez^®^ flow cell deposited on the sensing area; (iv) a PMMA cover including inlets and outlets pipes were connected to a Gilson^®^ 3 peristaltic pump to ensure continuous fluids flow of order of 190 μL/min, and (v) a HP8711C network analyzer to follow up phase and modulus variations versus time at a fixed frequency (See Supplementary Materials Figure S4).

Prior to the detection of the anlytes of interest, the SAW devices were cleaned with acetone and ethanol and then with a piranha solution (98% H_2_SO_4_/30% H_2_O_2_ 2:1 v/v) to remove possible traces of organic contaminants and to activate the gold surfaces [46]. The substrates were after that rinsed with ultra-pure water and ethanol, before being dried under ambient air. A 30 µl-drop of TDP-AN molecule was then deposited on the SAWs sensing area, by drop-cast, and the SAW device placed into an oven at 60 °C, to evaporate the chloroform solvent.

## 3. Results and Discussion

### 3.1. Analytical Modeling Using MATLAB Software

**Figure 1:**
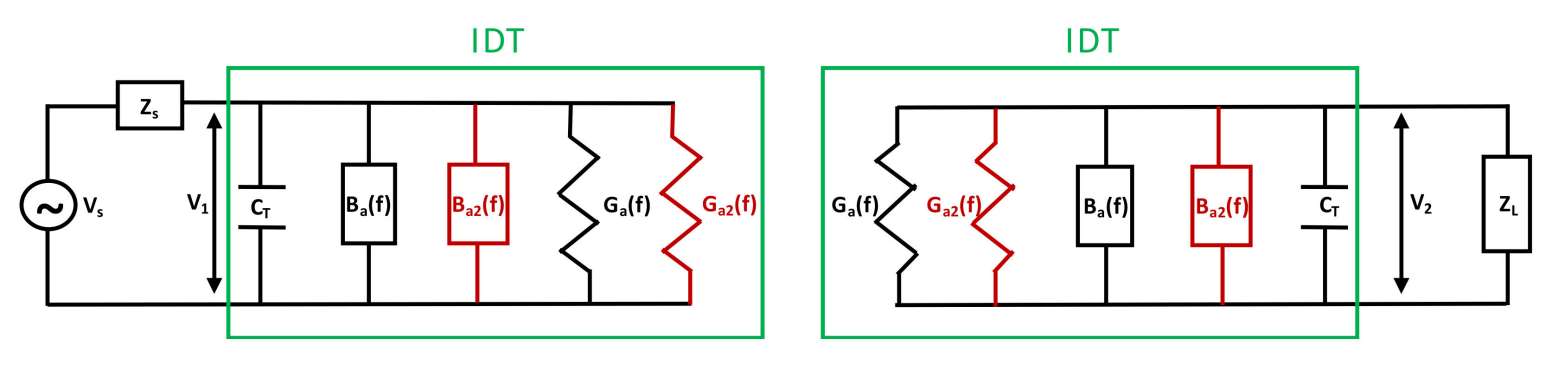
Schematic view of two equivalent circuits which takes into account both surface and volume acoustic waves.

The interest of MATLAB is to rapidly simulate the mathematical behavior of a SAW device in terms of transfer function. This can be done by considering an elementary equivalent electrical circuit, in which the operating frequency is related to the surface acoustic wave propagation. In this study, we have used the impulse response method which was developed by Hartmann, Bell, and Rosenfeld [47]. The Masson’s equivalent circuit is often used to represent the electrical behavior of basic interdigital transducers (IDT). To take fast volume waves into account, we proposed a second identical circuit in parallel as shown in Figure 1.

The equivalent circuit of IDT transducer consists of a total capacitance (C_T_) and two radiation admittances Y_ai_ for surface and volume acoustic waves (Equation (1)):

Y_ai_(f) = G_ai_(f) + j×B_ai_(f) (i = 1, 2) (1)

The real part G_ai_(f) represents the radiation conductance, while the imaginary one, B_ai_(f), is the acoustic susceptance determined from the Hilbert transform of the real part [47]. This proposed simple model allows the implementation of the corresponding mathematical equations in MATLAB software to quasi-instantaneously extract the system frequency response. MATLAB code has been successfully written and checked to perform the required analysis. Here, we considered a structure of 30 pairs of double fingers, operating at 104 MHz. Input parameters implemented in MATLAB code are gathered in Table 1.

**Figure 2:**
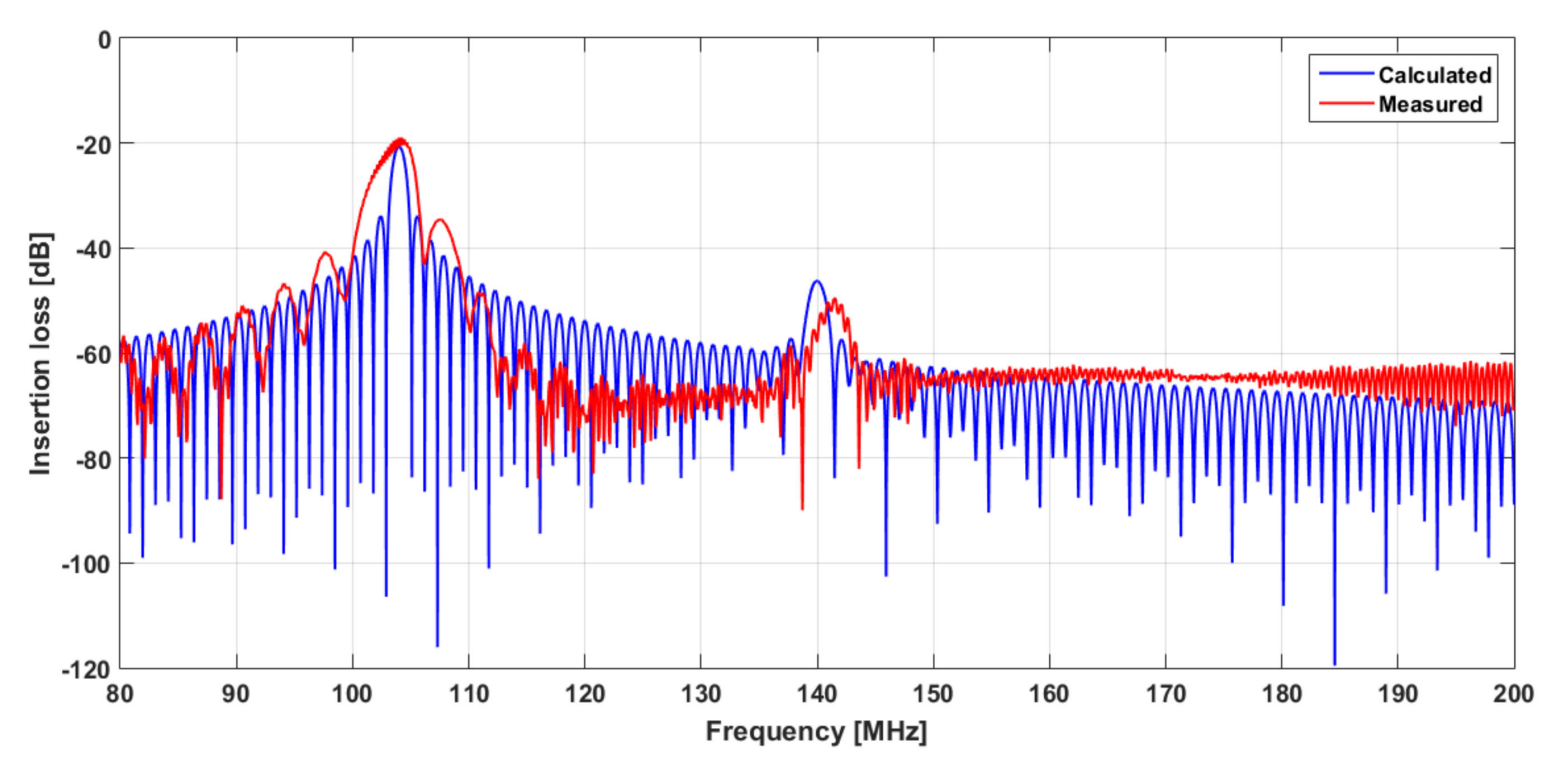
Calculated and measured insertion loss spectra of SAW delay line.

To assess the validity of this simplified model, a computation was done on a delay line configuration close to the real SAW device. Results, presented in Figure 2, show a good agreement between calculated and experimental S21 spectra, in terms of insertion loss (−19 dB measured against −20 dB calculated) and shape around the operating frequency (f_0_ =104 MHz). However, the bandwidth is slightly wider in the measured S21 spectrum than in the calculated one. This difference is probably due to the real losses in the piezoelectric material as well as to the imperfections in the real geometry of the IDTs (metallization rate, aperture, roughness, etc.), considered as ideal in the calculations.

For the value of bulk wave frequency (≈140 MHz), calculation can be adjusted to closely fit the experimental value, but at this stage, this is not essential, as our main objective is focused on surface acoustic waves.

From results presented in Figure 2, we clearly showed that MATLAB software can be used as a first approach to simulate the electrical behavior of a SAW delay line device. Nevertheless, this simulation does not consider several other parameters such as reflections between finger pairs, triple transit, and reflections at the edges of the substrate. Obviously, each of these parameters can be taken into account in the calculations, but this would make the model very complex. Alternatively, we considered a FEM simulation to overcome these limitations, as this method consider both physical and electrical properties of the piezoelectric substrate.

### 3.2. Numerical Simulation with COMSOL Multiphysics

**Figure 3:**
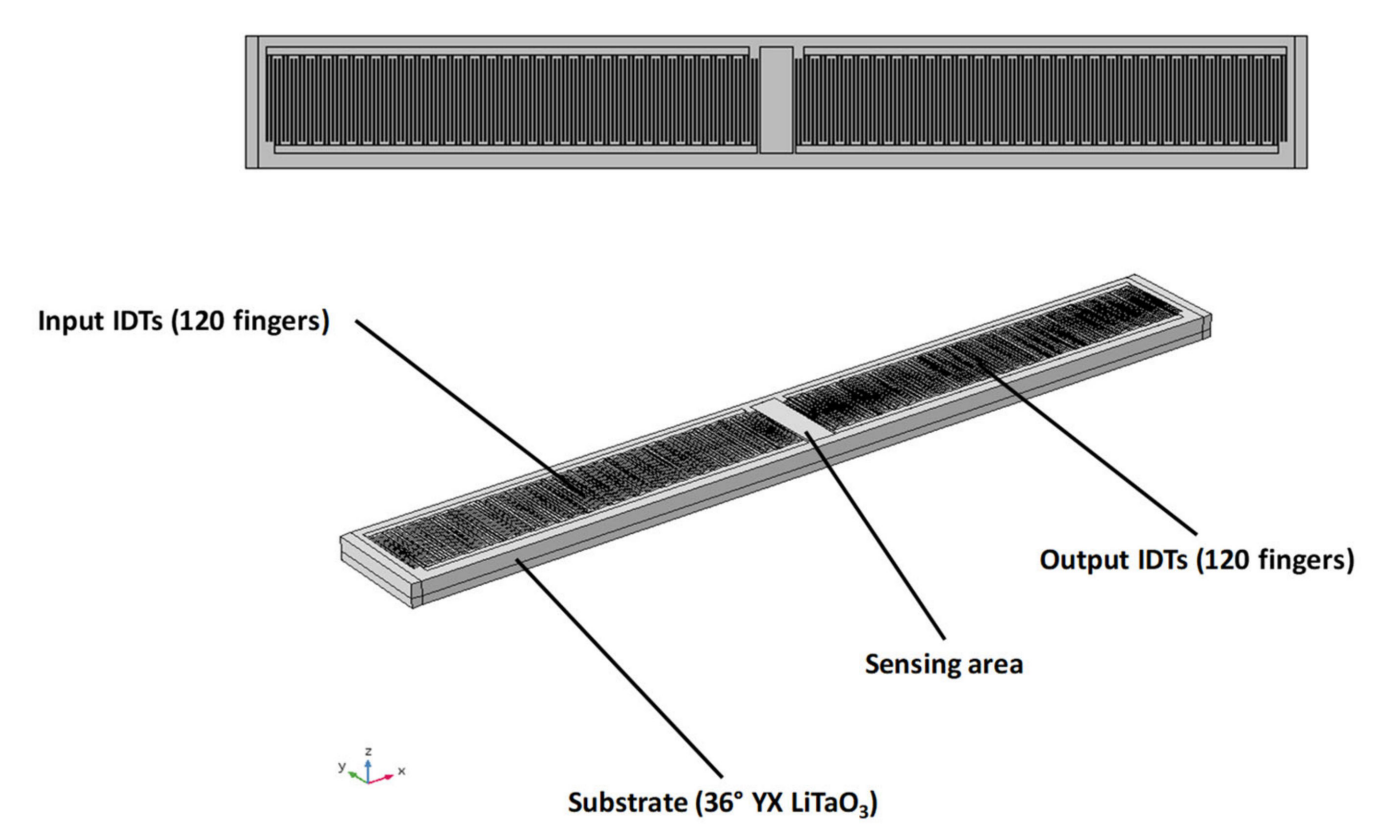
FEM 3D geometry of SAW device.

A COMSOL Multiphysics-based 3D model was created on 36 lithium tantalate (LiTaO_3_) piezoelectric substrate. Input and output IDTs (30 finger pairs each, fingers width = 5 μm) were in Cr/Au (20 nm/80 nm) with a periodicity of λ = 40 μm (i.e., operating frequency f_0_ = 104 MHz), and separated by a sensing area of 80 µm length (Figure 3). To save time, we chose to reduce the length of the sensing area instead of using large meshes.

**Figure 4:**
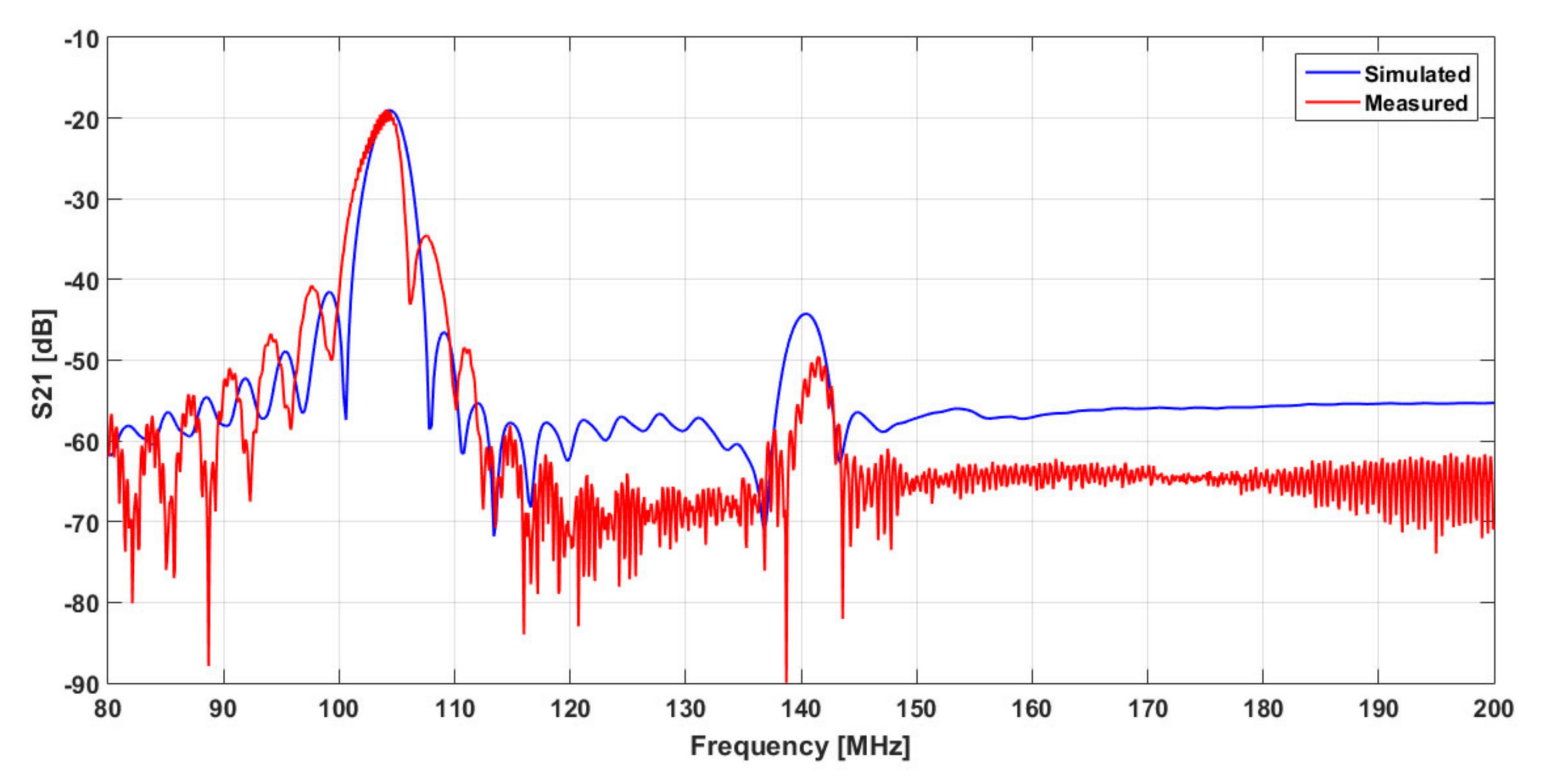
Simulated (3D model COMSOL, configuration: 30 pairs of double fingers + metallized sensing area of 80 µm length) and measured S21 spectra of SAW delay line.

The simulations were run from 80 MHz to 200 MHz with a frequency step of 100 kHz, and the obtained S21 spectrum was compared to the experimental one (Figure 4). Results show a good agreement between computations and measurements, in terms of shape, around the operating frequency (f_0_ ≈ 104 MHz), insertion loss values, and bandwidth. We also notice the presence of the expected “peak” around 140 MHz, characteristic of fast volume waves.

**Figure 5:**
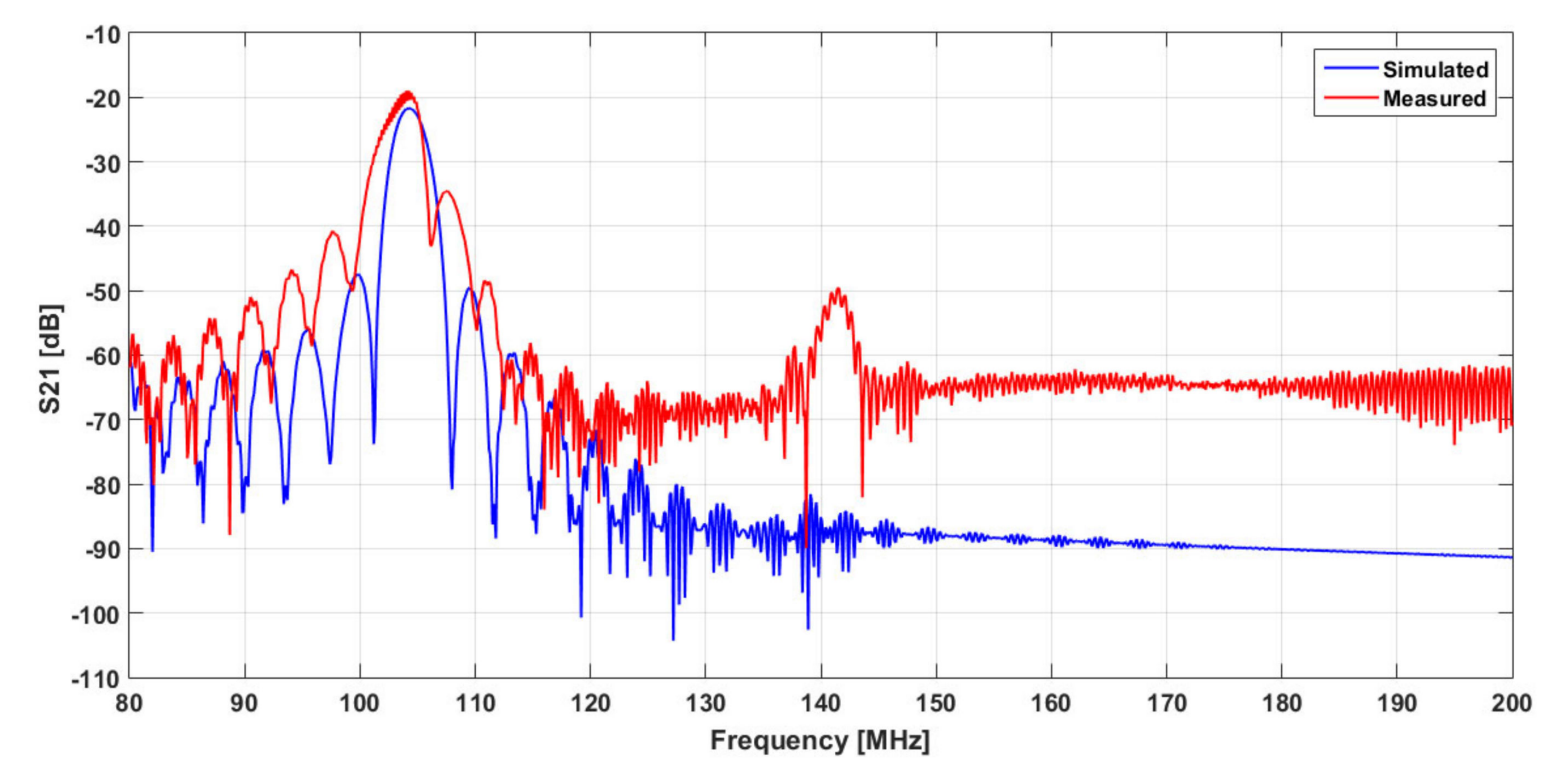
Simulated (2D model COMSOL of configuration: 30 pairs of double fingers + metallized sensitive area of 8 mm length) and measured S21 spectra of SAW delay line.

Even though the length of the sensing area has been drastically reduced (two orders of magnitude), this interesting 3D model remains time consuming (six weeks). A 2D model was thus investigated to reduce calculations durations. We first considered simpler structures, with two, then three pairs of double fingers, to assess the similarity between 2D and 3D models results (results not shown here). Once ensured of results concordance, we simulated a 2D structure with 30 pairs of fingers, having an 8 mm length sensing area, the same as the experimental device. The simulations were run from 80 MHz to 200 MHz with a frequency step of 100 kHz to access S21 parameter. Calculations duration was of the order of 14 h, largely inferior to that needed for a 3D model. The comparison between calculation and experimental spectrum (Figure 5) highlights a good agreement between the two spectra in terms of gain and expected shape around 104 MHz. The disappearance of the characteristic frequency around 140 MHz, characteristic of bulk waves, can be attributed to the geometrical conditions, as BAWs detection is indirect (from backscattering). However, this is not limiting, as the main objective of this study is related to the investigation of the surface acoustic waves.

**Figure 6:**
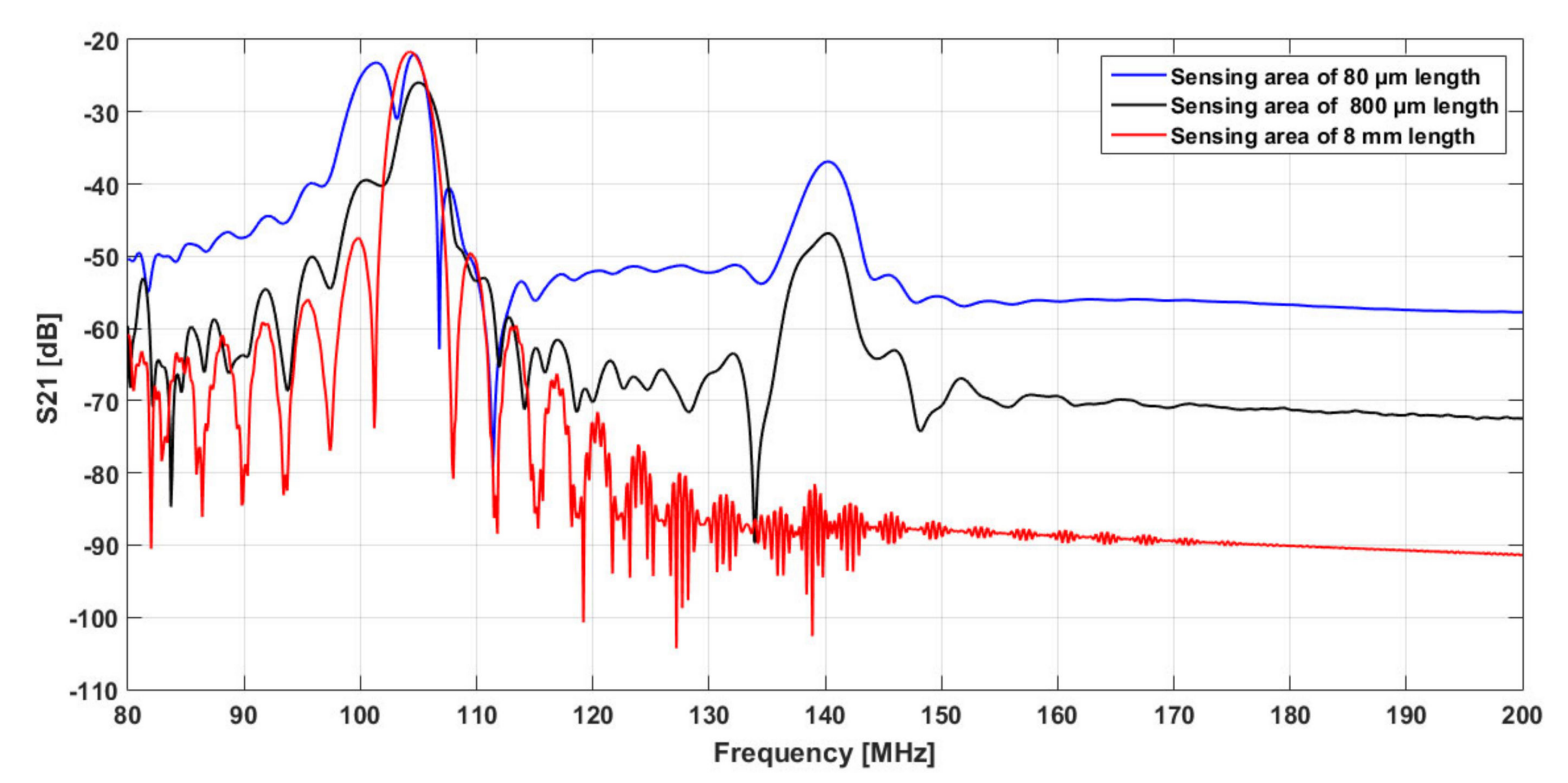
Simulated S21 spectra for different length of sensitive area.

Subsequently, and to save calculation time, we reduced the length of the sensing area by one then two orders of magnitude. Results presented in Figure 6 indicate that S21 spectrum corresponding to the extended area (8 mm), is the closest to the experimental one (regarding surface waves only). It is therefore this configuration that was thus considered for further investigations.

On the basis of the whole comparison results, we have made the choice of considering 2D simulation to accurately study the response of a 208 MHz SAW device and to check the sensitivity enhancement with frequency.

### 3.3. SAW Device Sensitivity Enhancement with Operating Frequency Increase

To design a 208 MHz-SAW-2D delay line, we have considered the same LiTaO_3_ piezoelectric substrate. Input and output IDTs (30 finger pairs each, fingers width = 2.5 μm) were designed in Cr/Au (20 nm/80 nm) with a periodicity of λ = 20 μm. A metallized Cr/Au (20 nm/80 nm) sensing area of 8 mm length separates the input and output IDTs. The simulations were run from 140 MHz to 260 MHz with a frequency step of 100 kHz. The corresponding calculated spectrum will be compared to experimental one (after the design of 208 MHz-SAW) at the end of this section.

**Figure 7:**
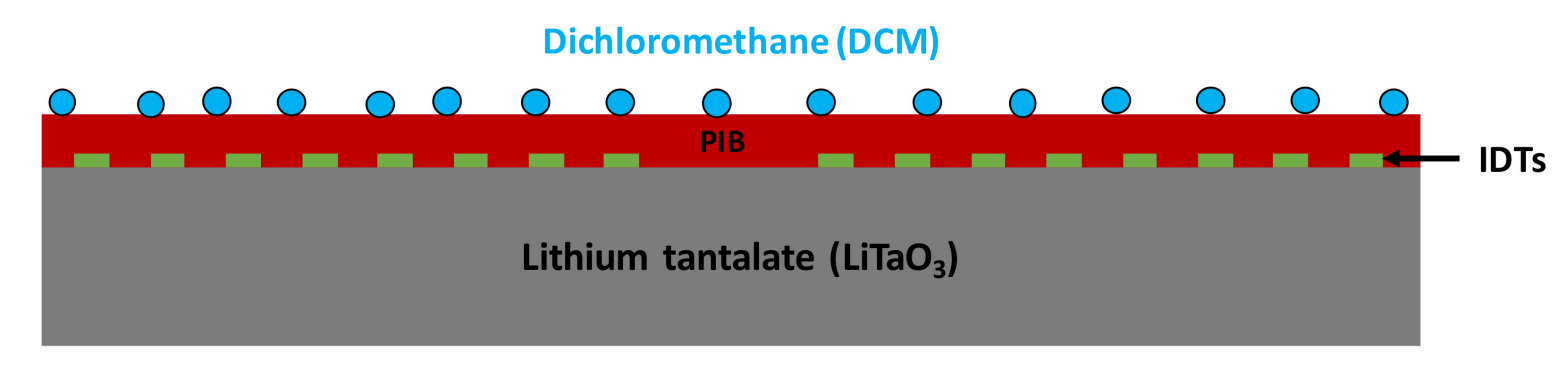
Schematic representation of a SAW device functionalized with a PCB layer for DCM recognition.

To compare the sensitivities of 104 MHz and 208 MHz chemical sensors, we designed 2D chemical sensors in which a 500 nm thick layer of polyisobutylene (PIB) was deposited on the entire surface of each delay line. PIB plays the role of a dichloromethane (DCM) recognition layer (Figure 7). Sensitivities were compared in terms of frequency shifts according to DCM concentration.

**Figure 8:**
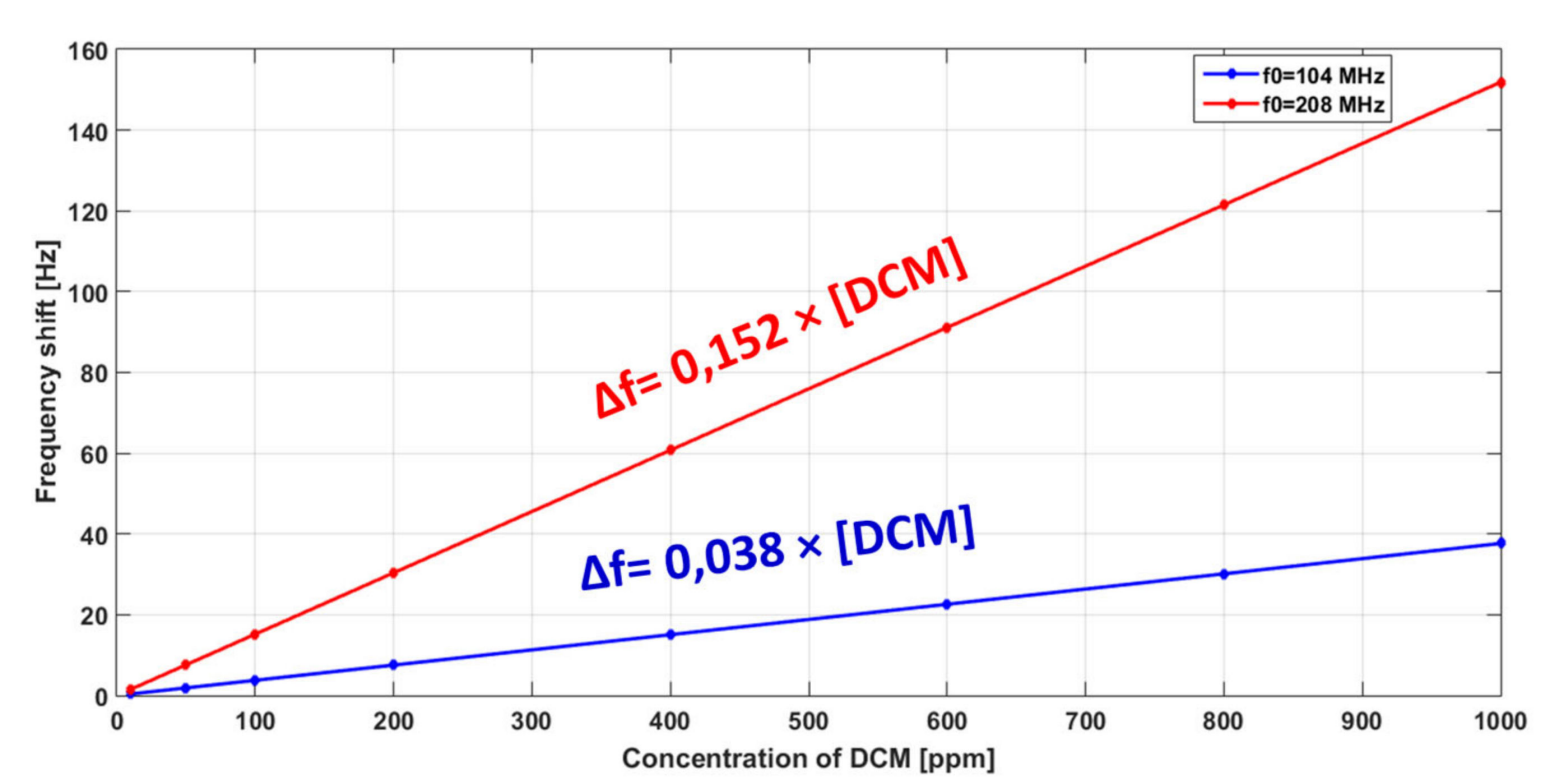
Sensitivity curves for 104 and 208 MHz SAW gas sensors.

Both SAW sensors were exposed to various concentrations of DCM gas ranging from 10 to 1000 ppm at atmospheric pressure and room temperature. DCM adsorption on the PIB layer leads to an increase of the sensing film density, and thus to a decrease of the characteristic frequency, as it is proportional to the adsorbed DCM concentration. The sensitivities of the sensors, estimated from the 2D calculated calibration curves, were of order of 38 mHz/ppm and 152 mHz/ppm for 104 MHz and 208 MHz sensors, respectively (Figure 8). Notice that only DCM molecules, “adsorbed on” PIB, were considered in this approach. The enhancement of sensitivity by a factor of four, in line with theory, is encouraging enough to allow consideration of the design and construction of 208 MHz sensors.

**Figure 9:**
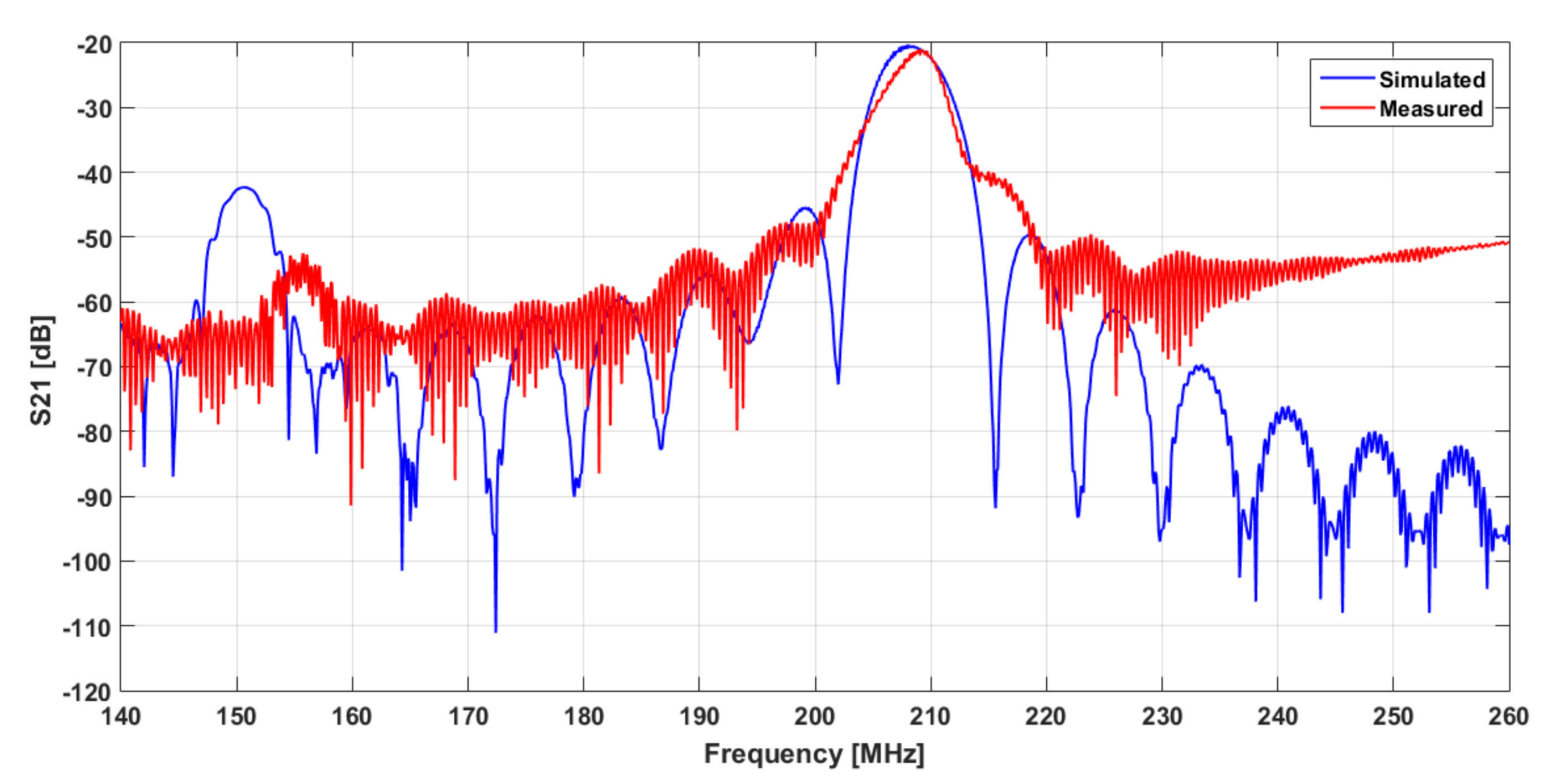
Simulated (2D model COMSOL, configuration: 30 pairs of double fingers + metallized sensitive area of 8 mm length) and measured S21 spectra of SAW delay line.

Based on these computational results, a 208 MHz SAW device was realized. Comparison between simulated and experimental spectra is presented in Figure 9. Results show a good agreement between measurements and simulations, in terms of expected shape around the operating frequency, (f_0_ ≈ 208 MHz). The ultimate goal of the previous part was to show that a 2D COMSOL Mutiphysics configuration permits to obtain all relevant parameters with very substantial time saving. Only the gas medium modeling was therefore done. The main application of SH-SAW sensors remains however the detection of analytes in liquid media. In this study, we made the choice to design 104 and 208 MHz devices for the detection of zinc ions in aqueous solutions.

### 3.4. Design of TDP-AN SAW Sensors

#### 3.4.1. Functionalization Step

**Figure 10:**
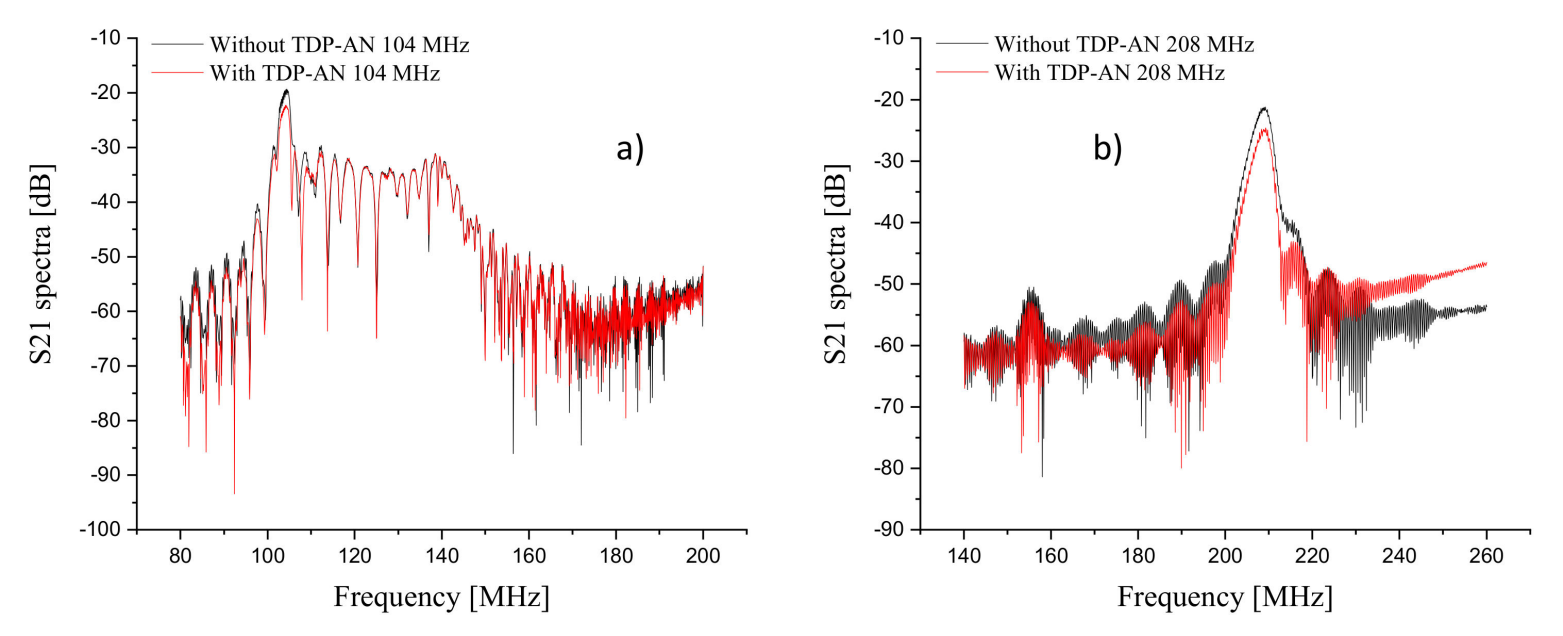
S21 spectra before and after the sensing area functionalization with TDP-AN molecule (**a**) for 104 MHz SAW; (**b**) for 208 MHz SAW.

The key step for the design of a chemical or biological sensor is the success of the sensing area functionalization step. Here, we have recorded the S21 spectra before and after TDP-AN drop cast on the sensing area of both 104 and 208 MHz SAW sensors (Figure 10).

The functionalization causes a decrease of order of 3.5 dB and 3.9 dB of the transfer function modulus for 104 and 208 MHz sensors, respectively, confirming the successful adhesion of TDP-AN on the sensing areas.

#### 3.4.2. Zn^2+^ Detection

**Figure 11:**
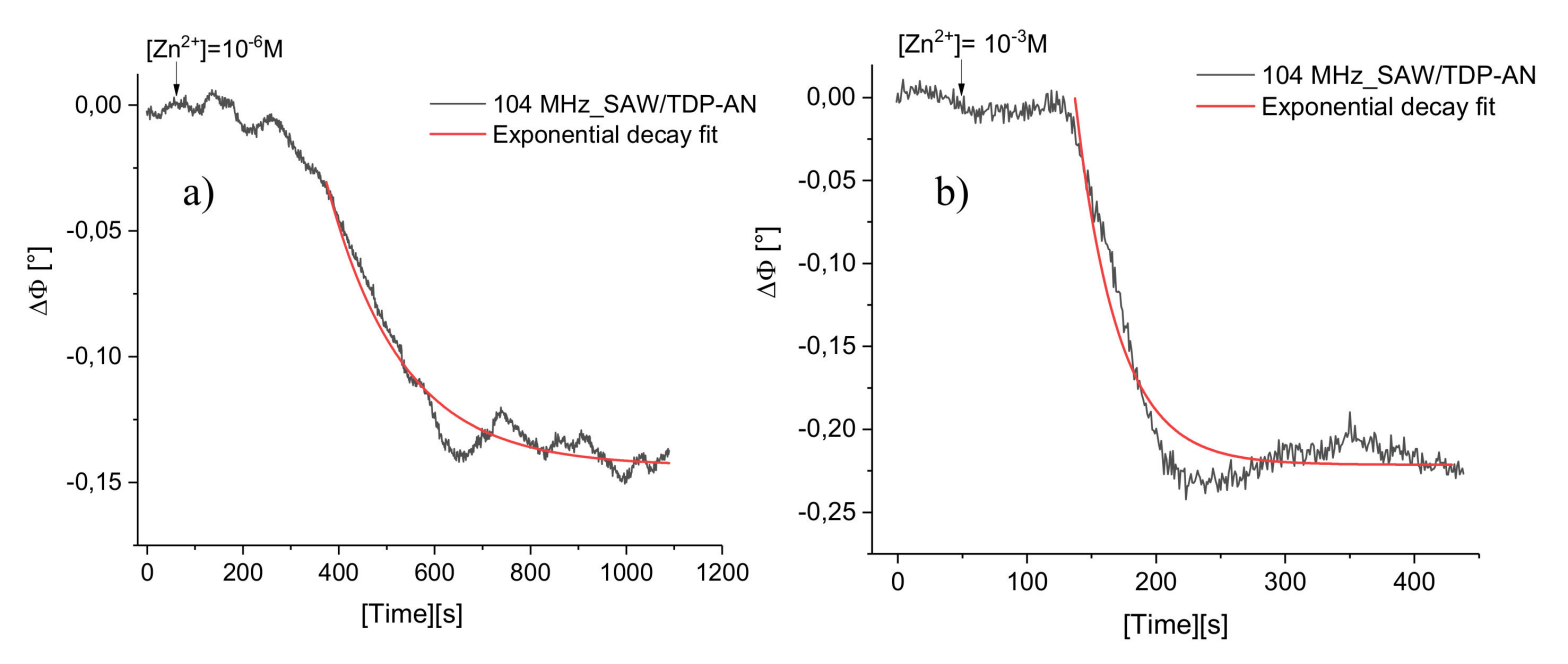
Phase-shift (∆Φ) variations versus time for 104 MHz_SAW/TDP-AN sensor after the injection of a zinc ion solution at a concentration of (**a**) 10^−6^ M; (**b**) 10^−3^ M.

The monitoring of zinc ions detection was investigated by following up the temporal variation of phase shift, for a fixed operating frequency. Prior to ions injection, a constant flow of double ionized (DI) water was brought, at a constant rate of 190 µL/min, on the sensing area of each SAW sensor. Once the stabilization reached, 10 µL of zinc ions solution of given concentration was injected into the fluidic circuit. This leads to a decrease of the phase output signal, indicating that TDP-AN molecule recognizes the analyte. Here, we have chosen to present the phase shift variations as a function of time after the injection of 10^−6^ M and 10^−3^ M of zinc ions for 104 MHz SAW sensor. The typical gravimetric responses are reported on Figure 11.

To provide information on the kinetic of interaction between TDP-AN and Zn^2+^ ions, Figure 11a,b were fitted with an exponential decay function. The corresponding time constants were of order of τ_[Zn_^2+^_]_ = 10^−6^ M = (157.05 ± 2.76) s and τ_[Zn_^2+^_]_ = 10^−3^ M = (32.95 ± 1.14) s. As expected, the characteristic time constant following the Zn^2+^ ions injection at a concentration of 10^−6^ M is higher than that obtained after adding of 10^−3^ M of zinc ion solution. Difference of the τ values after the two considered concentrations was also observed in the case of 208 MHz_SAW/TDP-AN sensor (Figures are not shown here).

**Figure 12:**
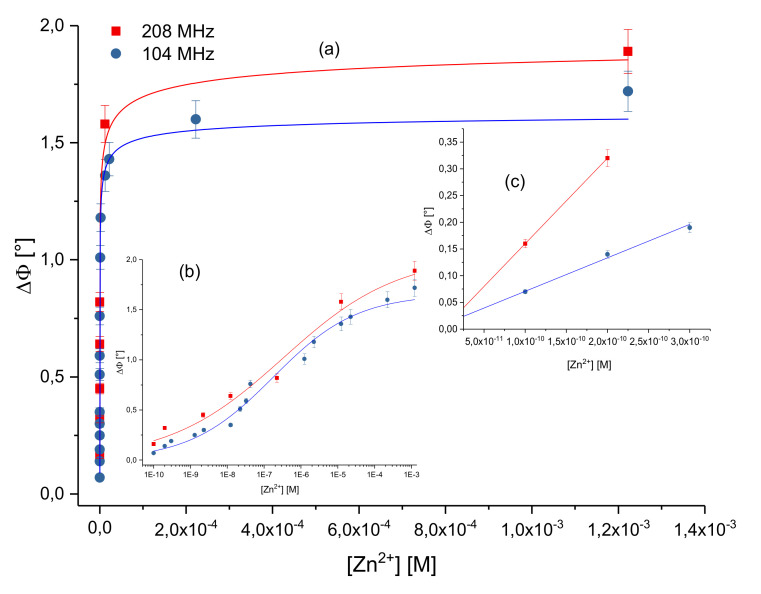
Phase-shift variations (ΔΦ) versus cumulative Zn^2+^ concentration for 104 and 208 MHz SAW sensors functionalized with TDP-AN molecules (**a**) in linear scale; (**b**) in logarithmic scale; the red and blue lines correspond to the best fit of experimental data according to the Hill model. (**c**) Slope at the origin of phase/concentration curves for both 104 and 208 MHz SAW sensors.

Phase-shift variations (ΔΦ) versus cumulative Zn^2+^ concentration for 104 and 208 MHz sensors are plotted in Figure 12. Each calibration curve was obtained by averaging three successive experiments. Results were compared in terms of limit of detection (LOD), dissociation constant K_d_, and sensitivity.

Limit of detection (LOD) is usually calculated from the signal to noise ratio. Here, we defined it as the lowest concentration detected by each functionalized SAW sensor. Gravimetric results indicate that the LOD was of about 0.1 nM for both sensors. Results, presented in Figure 12a, also indicate that the phase shift value at saturation ∆Φs (208 MHz) is slightly superior to that of ∆Φs (104 MHz), showing that increasing of operating frequency, from 104 MHz to 208 MHz, permitted to increase the output signal.

Dissociation constant (K_d_) is among the most important parameters in chemical sensing, as it provides an idea about the degree of affinity between a recognition layer and the further analyte. Here, we have chosen to fit the calibration curves with a Hill model in order to estimate the K_d_ value:

(2) $$\Delta \Phi \left(C\right)=\frac{A\times C^{\alpha }}{K_{d}^{\alpha }+C^{\alpha }}$$

∆Φ(C) corresponds to the phase-shift variations for a given Zn^2+^ concentration (C); K_d_ is the dissociation constant; A is an empiric constant, which is equal to ∆Φ_max_, i.e., at saturation; and α is the Hill coefficient.

Dissociation constants were estimated at: (2.8 ± 1.0) × 10^−7^ M and (3.4 ± 2.7) × 10^−7^ M for 104 and 208 MHz, respectively. These results exhibit comparable values for K_d_, within uncertainties, showing no effect of frequency as expected. Indeed, K_d_ constant is related to the interaction between TDP-AN molecule and Zn^2+^ ions and is therefore independent of the sensing method. Hill coefficient was of order of (0.36 ± 0.042) and (0.27 ± 0.03) for 104 and 208 MHz sensors, respectively, indicating negative cooperative interactions between TDP-AN and the binding sites [48].

Because of the non-linearity of the response of both sensors, their sensitivities were estimated from the slope at the origin of their respective calibration curves (Figure 12c). The obtained values were of the order of (7.0 ± 2.8) × 10^8^ [°/M] and (16.0 ± 7.6) × 10^8^ [°/M] for 104 MHz and 208 MHz sensors, respectively, indicating that the SAW sensitivity increases with frequency. The (S_208_ MHz/S_104_ MHz) experimental ratio was of order of 2.3, a value which is inferior to the theoretical one (equal to 4) [49]. These unbalanced values (2.3 and 4) indicate that the mass-loading is not the unique element which contributes to a SAW response. However, this increase in sensitivity augurs success for future applications intended to quantify very small quantities of pollutants in water.

## 4. Conclusions

MATLAB software is used as simulation tool to get access to the frequency response (S21 spectrum) of SAW delay line, with reasonable calculation time. By using the impulse response model where we have considered both surface and fast volume waves, instead of the former only as is usually the case, we achieve a good agreement between calculation and experiment. A full characterization, including second order effects (multiple reflections, interferences…) were then investigated by FEM simulations using COMSOL Multiphysics software. 3D simulations were in line with experiments, highlighting the robustness and accuracy of the considered model. Notwithstanding, and to significantly reduce the computation time, 2D models were built and compared to the 3D one. Results clearly shown that it is possible to accurately obtain all pertinent parameters with 2D simulations. The latter can thus be used with complete confidence to optimize the desired structures before any realization step. To evaluate the increase in sensitivity with the operating frequency, the 104 MHz and 208 MHz delay lines were modified with a polyisobutylene (PIB) to become sensitive to dichloromethane (DCM) gas. Simulations showed that sensitivity value was increased by a factor of four, in accordance with the theory, which encouraged us to develop a 208 MHz delay line in addition to the existing one (104 MHZ SAW sensor). The two realized devices were then tested as chem-sensors to detect zinc ions in solution and compared, mainly in terms of limit of detection (LOD), dissociation constants (K_d_), and sensitivity. For that, the 104 MHz and 208 MHz SAW sensors were functionalized with an anthracene derivate molecule (TDP-AN) as a sensing layer. As expected, the estimated values of LOD and K_d_ were comparable for both sensors. Gravimetric results indicate also that the increasing of operating frequency, from 104 MHz to 208 MHz, permitted to enhance the sensitivity by a factor of 2.3.

This work paves the way for the use of the TDP-AN/208 MHz SAW sensor in the Sarthe River Basin to detect heavy metal ions, especially zinc.

## Figures and Tables

**Table 1:** Input parameters implemented in MATLAB software to model a LiTaO_3_-104 MHz surface acoustic wave (SAW) device.

Parameters	Values
Electromechanical coupling coefficient (K^2^)	0.047
Surface wave velocity	4160 m·s^−1^
Fast bulk wave velocity	≈5600 m·s^−1^
Operating frequency (f_0_)	104 MHz
Number of finger pairs (N_p_)	30
Aperture (W_a_)	2000 μm
Length of delay line (D)	8010 μm
Source and load impedance (Z_s_ et Z_L_)	50 Ω
Capacitance per unit length for a pair of fingers (C_s_)	50 pF/m
Total static capacitance (C_T_)	6.04 pF

## Supplementary Materials

The following are available online at https://www.mdpi.com/1424-8220/20/17/4994/s1, Figure S1: Manufacturing process of SAW devices in LAUM’s clean room, Figure S2: Photos of the SAW sensor and further details of the interdigital transducers (IDTs), Figure S3: Details of the printed circuit board (PCB), Figure S4: Details of the fluidic system
