# Supplementary material for: Simulation/Experiment Confrontation, an Efficient Approach for Sensitive SAW Sensors Design
Source: Sensors (Basel). 2020 Sep 3;20(17):4994. doi: 10.3390/s20174994 (PMC7506909; doi:10.3390/s20174994)
Supplement: Supplementary file 1 [file sensors-20-04994-s001.pdf]

Supporting Information

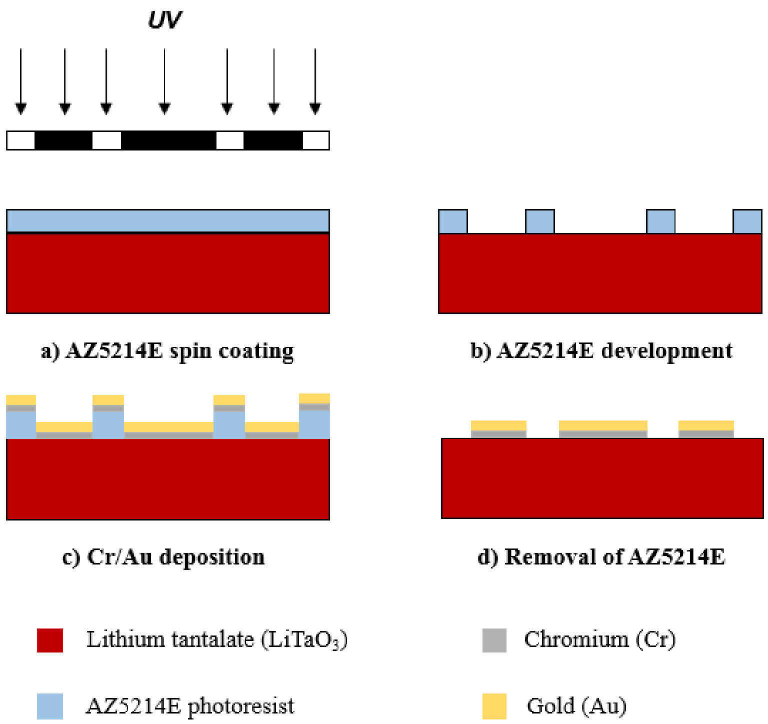

Figure S1. Manufacturing process of SAW devices in LAUM's clean room.

Prior to SAWs fabrication, the $LiTaO_3$ wafer was rinsed copiously with acetone and isopropanol, before being dried with nitrogen flow. The wafer was then placed into a vacuum oven, at 120 °C during 30 min, to remove surface moisture and dehydrate the surface of wafer. The AZ5214 photoresist was after that spin-coated at 2000 rpm for 8 s (Figure S1a). Subsequently, the wafer was placed on a hot plate at 90 °C for 1 min to evaporate the solvent of the resin. After exposition to ultraviolet radiation via a mask, the wafer was heated again at 110 °C for 45 s. The wafer was after that exposed to ultraviolet without mask, to reverse the photoresist, and then immersed in a bath of AZ726 MIF developer (Figure S1b). (20-nm Cr/80-nm Au) layers were after that deposited by sputtering (Figure S1c). The last step consists in immersing the samples in acetone bath to remove the AZ5214 photoresist (Figure S1d). Photography of the designed SAW device, manufactured in LAUM clean room, is presented in Figure S2.

Supporting Information

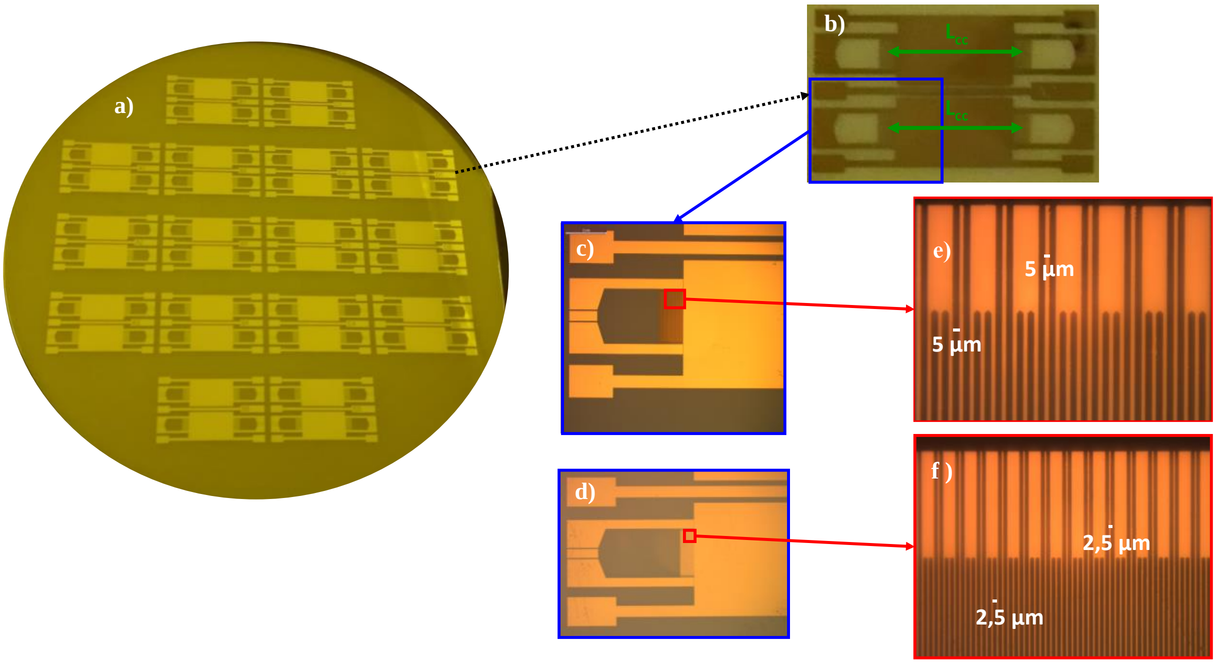

Figure S2. Photos of the SAW sensor and further details of the interdigital transducers (IDTs).

(**a**) Photography of a processed $LiTaO_3$ wafer with 16 SAW devices.

(**b**) Dual delay lines configuration: L_{cc}=9.2 mm for 104 MHz SAW sensors and L_{cc} = 8.6 mm for 208 MHz SAW sensors.

(**c**) Zoom on a 104 MHz SAW sensor's IDTs and further contact pads; d) zoom on the corresponding interdigitated combs (fingers width = 5 µm).

(**d**) Zoom on a 208 MHz SAW sensor's IDTs and further contact pads; f) zoom on the corresponding interdigitated combs (fingers width = 2.5 µm).

Supporting Information

Photo of the dual delay lines SAW-sensor

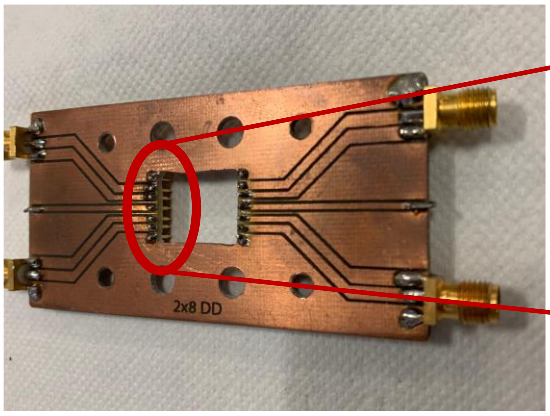

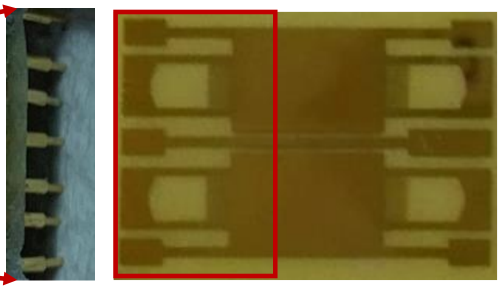

Zoom on the 7 pins which ensure the electrical contacts with the dual delay lines

Photo of the PCB with 4 SMA connectors

Figure S3. Details of the printed circuit board (PCB)

Supporting Information

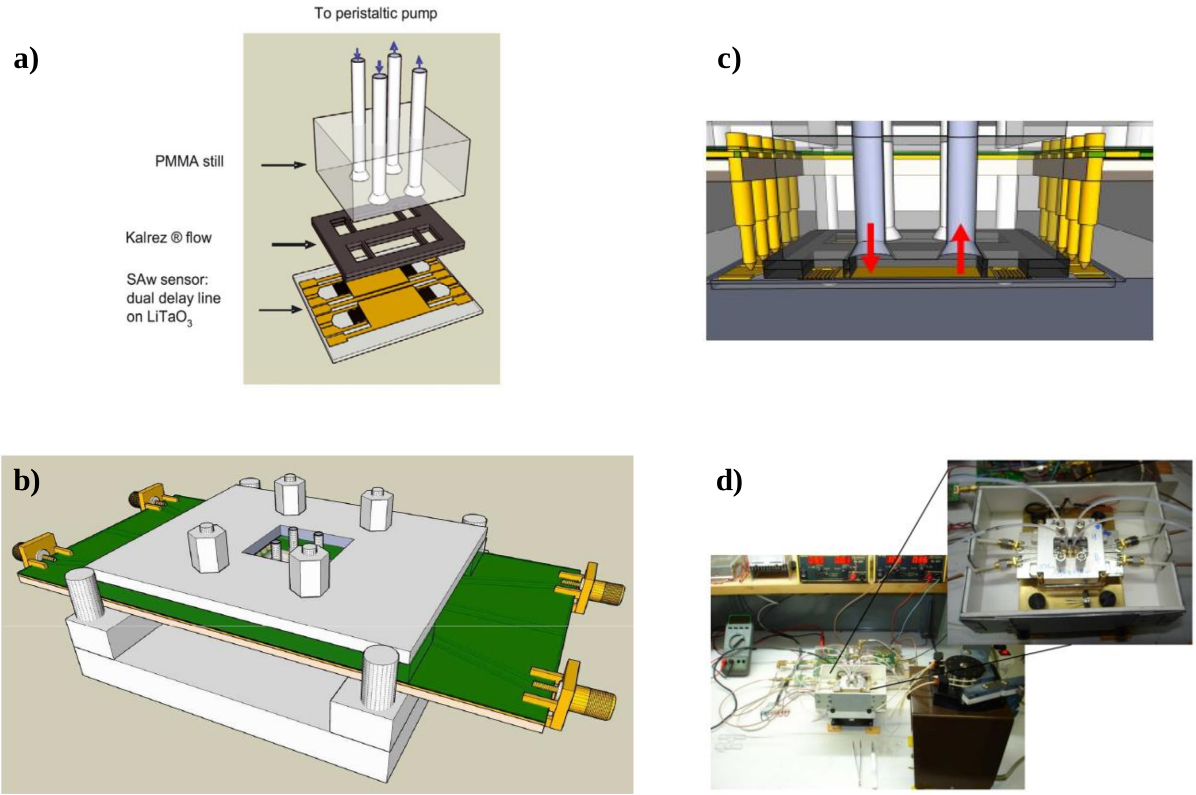

Figure S4. Details of the fluidic system

Schematic representation of: (**a**) the dual delay lines and details of fluidic cell; (**b**) the system assembly with printed circuit board; (**c**) Sectional view of the entire system with gold pins on electric contacts, Kalrez tank and inlet/outlet PMMA tips; (**d**) Overview of the system assembly and the fluidic circuit with the peristaltic pump.
